# Displacement Back Analysis for a High Slope of the Dagangshan Hydroelectric Power Station Based on BP Neural Network and Particle Swarm Optimization

**DOI:** 10.1155/2014/741323

**Published:** 2014-07-20

**Authors:** Zhengzhao Liang, Bin Gong, Chunan Tang, Yongbin Zhang, Tianhui Ma

**Affiliations:** Institute of Rock Instability and Seismicity Research, Dalian University of Technology, Dalian, Liaoning 116024, China

## Abstract

The right bank high slope of the Dagangshan Hydroelectric Power Station is located in complicated geological conditions with deep fractures and unloading cracks. How to obtain the mechanical parameters and then evaluate the safety of the slope are the key problems. This paper presented a displacement back analysis for the slope using an artificial neural network model (ANN) and particle swarm optimization model (PSO). A numerical model was established to simulate the displacement increment results, acquiring training data for the artificial neural network model. The backpropagation ANN model was used to establish a mapping function between the mechanical parameters and the monitoring displacements. The PSO model was applied to initialize the weights and thresholds of the backpropagation (BP) network model and determine suitable values of the mechanical parameters. Then the elastic moduli of the rock masses were obtained according to the monitoring displacement data at different excavation stages, and the BP neural network model was proved to be valid by comparing the measured displacements, the displacements predicted by the BP neural network model, and the numerical simulation using the back-analyzed parameters. The proposed model is useful for rock mechanical parameters determination and instability investigation of rock slopes.

## 1. Introduction

Mechanical parameters of rock masses are crucial for stability analysis of high rock slopes [1, 2]. They can be obtained through laboratory experiments or in situ tests. However, the mechanical parameters of rock specimens obtained in laboratory experiments cannot represent those parameters of rock masses. In addition, due to many types of fractures and joints at different scales existing in rock masses and complex geostress conditions, it is difficult to acquire the mechanical parameters exactly in in situ tests. Because in situ tests have many drawbacks such as poor reproducibility, long period, and high costs, field displacement measurement is widely applied in rock engineering projects.

Displacement back analysis has been widely used to derive rock mass mechanical parameters [3–6]. The methods used in the displacement back analysis of geotechnical engineering projects can be broadly divided into two types, namely, inverse method and optimization method. The inverse method, such as that suggested by Sakurai and Takeuchi [5], is the inverse of the common numerical simulation procedure to solve some of the material parameters or loading conditions based on observed displacements. Rapid numerical solution and a number of simplifying assumptions, including uniform media, uniform or linear stress filed, and one-step excavation, enhance the popularity of the inverse method. However, for some excavation engineering projects such as high slopes, the problems may be very large in scale involving multimaterial, and there is a complex initial geostress filed due to the multimedia and tectonic stress. Moreover, multisteps of excavations and supports will last a long period, inducing stress field variation during the construction process [6]. These characteristics limit applications of the inverse method in slope engineering.

The optimization method used the summed squared errors between the calculated displacements and their corresponding observed values as the objective function. The solution of the objective function is based on some optimization techniques for determining a set of material parameters or loading conditions that make the value of the objective function a minimum [6].

Low efficiency and low reliability are the two drawbacks of the optimization method. The numerical methods, such as finite element method or finite difference method, are often applied to calculate the stress and displacement of the model in parameter adjustment in the optimization of the objective function. To achieve the minimal value of the objective function, a great number of parameter adjustments are needed. Therefore, it is impossible to apply the routine optimization method to resolve large scale problems with a great number of freedom degrees because the solution by either the FEM or FDM could be very time consuming.

In addition, the objective function for back analysis could be multimodal, and the optimization results could depend on the initial values in some routine optimization methods such as the Powell method. The range of the mechanical parameters is unlimited theoretically, so its low efficiency makes it unsuitable for estimated parameter search.

Fortunately, the back-propagation neural network (BP network) provides a reliable method instead of the FEM or FDM calculation, establishing the high nonlinear function between the estimated parameters and the measured displacement. Furthermore, some useful optimization models such as genetic algorithm and particle swarm optimization method provide efficient methods to enhance the search speed to achieve reliable convergence solution.

Studies show that the intelligent back analysis method can be used to deal with the identification of rock mechanical parameters, build the nonlinear relationship among variables effectively, and overcome many defects of traditional optimization algorithms. Deng and Lee [6] proposed a novel method for displacement back analysis based on error back-propagation neural network and genetic algorithm in the slope stability analysis of the Three Gorges Project. Xia-ting et al. [7] used neural network model and genetic algorithm to estimate the mechanical rock mass parameters of the permanent shiplock at the Three Gorges Project.

These optimization algorithms should resolve global optimal solution in a fast convergence rate and determine the unknown parameters among a large variable space. It has become one of the most potential approaches in the displacement back analysis. Researchers have performed many investigations and achieved significant progress on the aspect of back analysis in geotechnical engineering. Liang et al. [8] proposed a back analysis model to estimate the elastic moduli of the high slope of the Three Gorges Project. Tan and Zheng [9] introduced Newton-Laplason iterative method into the back analysis, and they verified that strength parameters could be obtained inversely by using displacements of several monitoring points. Feng et al. [10] summarized several new methods for intelligent feedback analysis. However, because multipoint extensometers are often buried after each step of excavations, the deformation of rock masses induced by elastic strain energy release cannot be measured before the current excavation step. Therefore, how to analyze the measured deformation data and apply them in back analysis for safety evaluations become very important.

In this paper, the back-propagation neural network was used to construct a function between undetermined parameters and displacements of the rock mass of the right back slope in Dagangshan Hydroelectric Power Station project. Totally 32 training samples were created by numerical simulations using FLAC3D code according to orthogonal experimental design. The particle swarm optimization method was applied to initiate the training weights and search for satisfactory deformation parameters. By using the deformation parameters obtained by the back analysis as input parameters, the results of the numerical simulations agreed well with the measured displacements. The method of combination of the BP neural network and particle swarm optimization is proved to have a powerful capability of resolving deformation parameters in rock slope problems with complex geostress and multiexcavation steps.

## 2. Brief Description of the High Slope of the Dagangshan Hydroelectric Power Station

**Figure 1:**
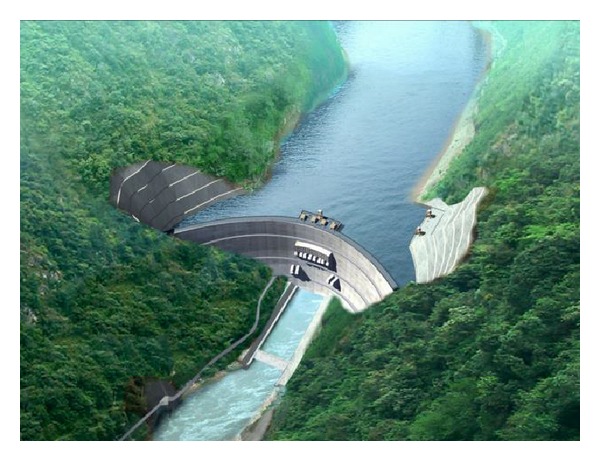
Survey of the Dagangshan Hydropower Station.

The Dagangshan Hydroelectric Power Station is located at the midstream of Dadu River in Shimian County, west of Sichuan Province in China. It is one of the largest hydroelectric projects which are currently being constructed along the mainstream of the Dadu River. The normal water level of the station dam is 1130 m, the dead water level is 1120 m, and the storage capacity below the normal water level is about 0.742 billion m^3^. Its maximum height is nearly 210 m. The power station is installed with a capacity of 2600 MW [11]. The dam is located in a typical valley of a “V” shape with steep and high bank slopes (Figure 1).

**Figure 2:**
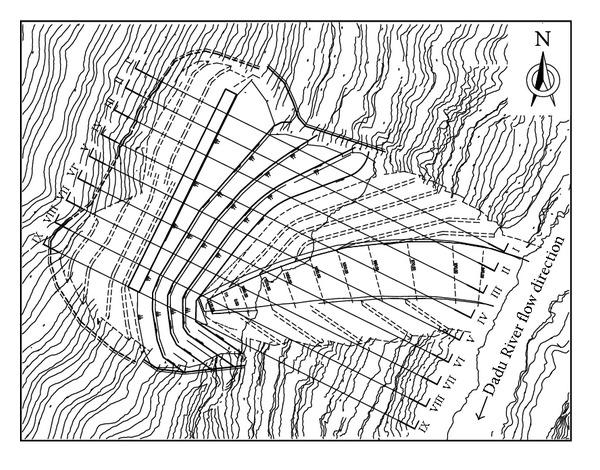
Layout of the right bank slope.

The dam area of Dagangshan Hydroelectric Power Station has complex geological conditions with deep fractures, developed faults, and unloading cracks. The rock mass in engineering area has formed a series of high and steep slopes caused by the excavations. As the strike of most weak structural surfaces is parallel to the river and the dip is steep, the problem of slope stability is rather serious. It mainly involves abutment high slope at right and left banks. The dip angle of the right bank slope varies from 35° to 40° above 1220 m and varies from 40° to 50° below 1220 m. The height of the slope is more than 600 m [11]. The topographic map of the right bank slope is shown in Figure 2.

The bed rock of slope is mainly composed of grey and reddish monzonitic granite, which is hard and core bread, rib spalling frequently occurs in unloading zones, and aplitic granite dykes as well as dolerite dykes are exposed on the natural slope surface in places. The faults mainly develop along with dolerite dykes with a steep reversal dip angle. Many dykes develop in the slope, such as *β*_5_, *γ*_L5_, *β*_202_, *β*_96_, *β*_4_, *β*_85_, *β*_62_, *β*_83_, *β*_68_,  *β*_117_, *β*_43_,  *β*_143_, and *β*_82_. Some structural planes also develop in the slope, such as fault *f*_231_, fault *f*_208_, and some unloading fissures named XL_9-15_ and XL_316-1_. The right bank slope of the Dagangshan Hydroelectric Power Station with complicated geological conditions is very steep, and unloading fissures and faults with steep dip angles have considerable influence on the slope stability, which has rarely been observed in other hydroelectric projects [12–16].

According to the geological survey data, approximately seventy-eight dolerite dykes (*β*) and eight aplitic granite dykes (*γ*_L_) develop in the right bank slope, such as *β*_4_, *β*_97_(*f*_93_), and *β*_146_. Approximately eighty-four faults with steep reversal dip angles develop along with dolerite dykes containing rock blocks, microfragments, and mud.

The geological survey also indicates that intensive open cracks can be observed inside the slope with a distance of 100 m from the slope surface. Two main unloading fissures named XL_9-15_ and XL_316-1_ are most concerned, which will control the displacement and stability of the slope. Further investigation has shown that the stability of the right bank slope is controlled by a potentially instable block whose bottom slip surface is formed by weak structural planes dipping outside the slope (such as *f*_231_ and *f*_208_) and rigid structural planes (such as XL_9-15_ and XL_316-1_) and back cutting surface is formed by dyke fracture zones (*β*_5_(*F*_1_), *γ*_L5_, and *γ*_L6_) [17].

## 3. Back Analysis Model

### 3.1. BP Neural Network

Artificial neural network can be seen as a set of parallel processing elements, and the suitable mathematical methods can be used to change the weights and thresholds to perform specific functions. The BP neural network can figure out each layer's error derivatives by using the back-propagation algorithm according to the generated weight matrices and threshold matrices. And then, BP adjusts corresponding matrices on the basis of error derivatives and square error sum to approach the mapping relation between the system input variables and output variables step by step.

**Figure 3:**
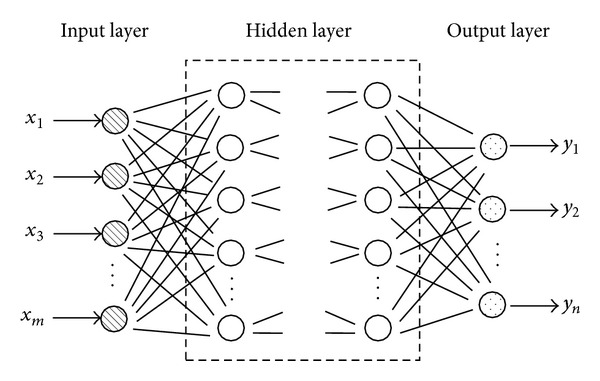
Typical structure of a BP neural network.

The typical structure of a BP neural network is shown in Figure 3. It has one input layer, one or more hidden layers, and one output layer, with each layer consisting of one or more neurons.

The number of neurons (*m*) in the input layer is the same as the number of mechanical parameters to be solved, and the number of neurons (*n*) in the output layer is the number of the measured displacements. Usually, only one hidden layer is needed. The number of neurons (*p*) in the hidden layer can be specified either manually or by an optimization method [6]. The training samples are often used to adjust the weight values by making the summed squared error between the displacements from numerical simulation and those from BP network a minimum. For the training samples, the input parameters can be prepared by the parameter experiment design method, while the corresponding output parameters can be prepared by numerical simulation.

**Figure 4:**
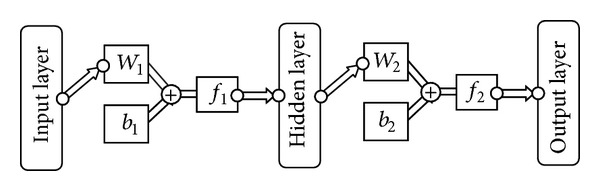
Flow chart of BP neural network.

The calculating procedure of a three-layer BP neural network is shown in Figure 4. *W*_1_ and *b*_1_ are weight matrix and threshold matrix between the input layer and the hidden layer, respectively; *W*_2_ and *b*_2_ are weight matrix and threshold matrix between the hidden layer and the output layer, respectively; function *f* is the transfer function between two adjacent layers. Three transfer functions, including tan-sigmoid transfer function (tansig), log-sigmoid transfer function (logsig), and linear transfer function (purelin), are the most commonly used transfer functions for multilayer networks.

However, the summed squared error between the displacements from numerical simulation and those from BP network depends on the randomly generated initial weights and the thresholds, so it is easy to fall into local convergence in the training process. Therefore, an improved particle swarm optimization algorithm with global optimization capability is necessary for the initiation of the weights and the thresholds of the BP neural network.

### 3.2. Particle Swarm Optimization

The particle swarm optimization (PSO), as an important branch of evolutionary algorithm, is a computational method that optimizes a problem by iteratively trying to improve a candidate solution with regard to a given measure of quality [18]. PSO avoids complex genetic operators and takes advantage of cooperation and competition. Each particle of the swarm represents one candidate solution of the problem being optimized. The quality of solution is evaluated by defining fitness function. Compared with other optimization algorithms, the superiority of PSO lies in fast convergence rate, simple program structure, and less calculation parameters. In order to further improve the convergence velocity and accuracy, some scholars [19, 20] adopt the dynamic inertia weight **w** that linearly decreases with iterative generation increasing, which ensures the good global searching capacity of PSO in the optimization and enhances the convergence performance. In this paper, the improved particle swarm optimization algorithm is used.

On the one hand, particle swarm optimization is used to initialize the weights and thresholds of BP neural network, solve the problem of local convergence, and guarantee the correctness of the network training in this paper. On the other hand, PSO is adopted to determine suitable values of the rock mechanical parameters within the ranges as an important part during the process of back analysis.

### 3.3. Back Analysis Procedure

Generally, engineering geological conditions and rock characteristics cannot be quantified completely, and relation function between rock property parameters is highly nonlinear, so that it is difficult to describe the complex mapping by using a determined mathematical model. Artificial neural network is especially suitable for the situation that there is no formula between parameter variables and objective function values [21]. This study adopted a BP neural network to establish the nonlinear mapping function between the deformation parameters and incremental displacements. Only the parameters of the elastic moduli are considered in this study.

**Figure 5:**
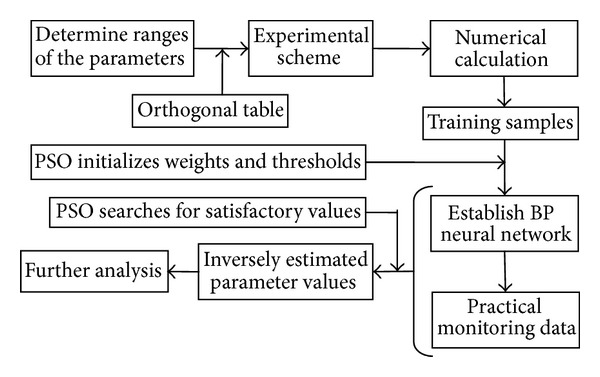
Back analysis flow chart based on BP neural network optimized by PSO.

The flow chart of the back analysis based on BP neural network optimized by PSO is shown in Figure 5. Firstly, the distribution ranges of elastic moduli of the high rock masses at the right bank of the Dagangshan Hydroelectric Power Station were determined by analyzing the geological conditions, the laboratory and field test results, and the practical monitoring data. Secondly, the training samples were created by calculating the incremental displacements on the basis of orthogonal experimental design. Thirdly, the samples were used to train the BP neural network which was initialized by PSO in advance. Thus, the nonlinear mapping relation between the elastic moduli of the rock masses and incremental displacements was established. The measured displacements in the slope were input into the established BP neural network to search the most optimized parameters using the particle swarm optimization model, which made the summed square error between the simulated displacements and the measured ones a minimum. Finally, the elastic moduli were determined to carry out further stability analysis of the slope.

## 4. Results of Monitoring Displacement

**Figure 6:**
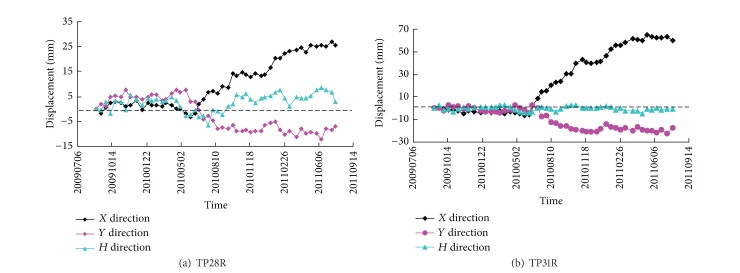
Accumulative displacement curves of the monitoring points TP28R and TP31R.

Displacement monitoring provides a helpful technique to predict slope stability. Trends of displacement variation can be traced during excavation and supporting periods. The excavation of the right bank slope of Dagangshan Hydroelectric Power Station caused lots of concrete surface cracks due to stress adjustments caused by the excavations. The monitoring displacement data of points TP28R and TP31R, which were located at Elevation 1100 m and Elevation 1070 m, are shown in Figure 6. It can be observed that the slope has large displacement obviously along the horizontal direction (*X* direction) under the excavations.

**Figure 7:**
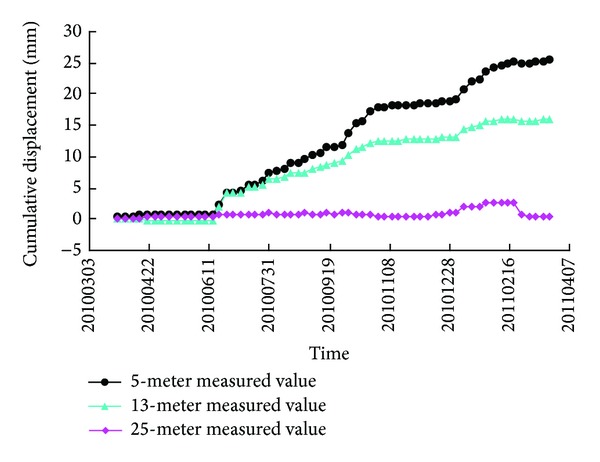
Accumulative displacement curves of M^4^_10RBP_.

A number of multipoint extensometers were embedded in the right bank slope at different locations to monitor the inside displacements. The monitoring results of the multipoint extensometer M^4^_10RBP_ at EL 1135 m are shown in Figure 7. The distances of these three monitoring points of M^4^_10RBP_ to the excavation face are 5 meters, 13 meters, and 25 meters, respectively.

**Figure 8:**
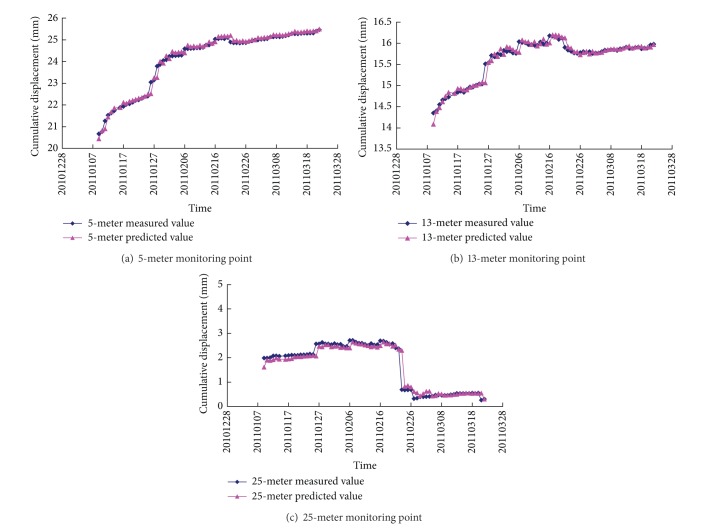
Displacement comparison between the measured displacements and the predicted displacements.

Usually, a three-layer BP neural network with *M* nodes in input layer, 2*M* + 1 nodes in hidden layer, and *N* nodes in output layer can be trained to express any functional relationship accurately [22]. Therefore, a BP neural network model with 10 input layer nodes and 1 output layer nodes was supposed to predict the elastic moduli of the rock masses of the high slope. The node number of the hidden layer was assigned to be 21 and the structure of the BP neural network model is 10-21-1. The BP neural network model was trained by using the samples created with the data of M^4^_10RBP_ monitored from March 27, 2010, to January 9, 2011. Then, other data were used for testing the validity of the BP neural network model. The comparison between measured displacements of M^4^_10RBP_ and the predicted displacements by the BP neural network is shown in Figure 8. It can be found that the proposed model can reflect the deformation trend with a good accuracy and basically meet the practical requirement in the slope engineering project.

## 5. Displacement Back Analysis

### 5.1. Material Properties Used in the Simulations

**Figure 9:**
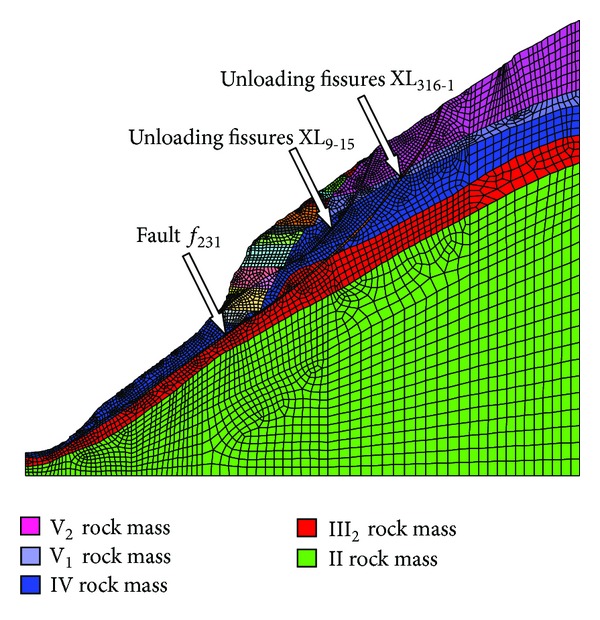
Numerical model of section IX-IX of the right bank slope.

According to the geologic investigations and monitoring data, Profile LPIX-IX (0+161.19 from upstream to downstream) was selected as the typical profile for displacement back analysis. The model covered most of the disturbed zones of the high slope: 700 meters long toward the inner slope from the centerline of the riverbed in transverse direction (as *X* direction) and 625 meters in vertical direction from EL 900 m to EL 1525 m (as *Y* direction). Only a unit thickness in *Z* direction was considered by simplifying the model into a plan strain problem. The bottom boundary was fixed in the vertical direction and surrounding boundaries were fixed in their respective normal directions. The number of the numerical elements is 4923, and the number of nodes is 10244. All the monitoring points were located at the nodes. The weak geological structural planes were built in the model, namely, fault *f*_231_, fault *f*_65_, and unloading fractures such as XL_9-15_ and XL_316-1_. The mesh of the right bank slope is shown in Figure 9.

According to the weathering degree, the rock masses in the right bank slope of the Dagangshan Hydropower Station were divided into six grades as follows: (a) completely weathered rock (V_2_); (b) intensely weathered rock (V_1_); (c) heavily weathered rock (IV); (d) upper part of moderately weathered rock (III_2_); (e) lower part of moderately weathered rock (III_1_); and (f) slightly weathered and fresh rock (II).

According to the results of the field tests, laboratory tests, and related practical engineering experience [23, 24], the details of the material properties used in the model are listed in Table 1. There parameters include the density (*g*), elastic modulus (*E*), cohesion (*c*), uniaxial compressive strength (*σ*_*c*_), Poisson ratio (*μ*), and the inner friction angle (*ϕ*).

### 5.2. Measured Incremental Displacements

The excavation of the right bank slope started in November 2007, and the excavations down to EL 1135 m were all finished in December 2008. The slope under the dam top was excavated to EL 1100 m in April 2009.

**Figure 10:**
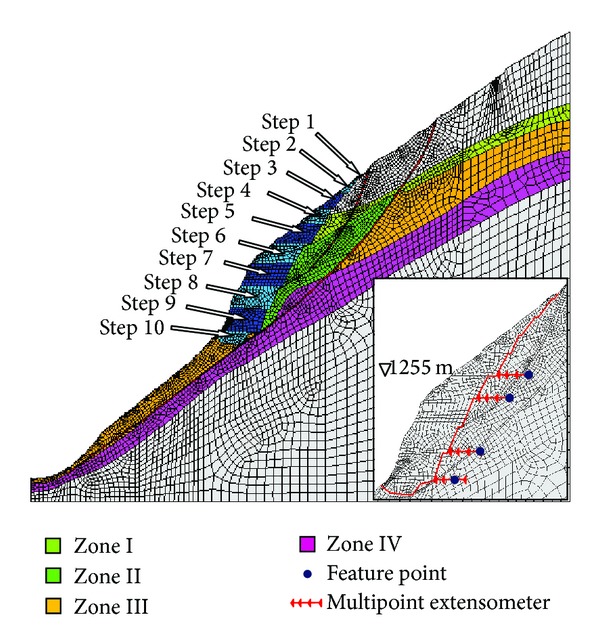
Monitoring points and excavation steps in the slope.

The multipoint extensometers were installed in three major locations to monitor the slope deformation. The excavations are divided into 10 steps as shown in Figure 10.

Monitoring data from several geological points are generally selected for back analysis. Because the multipoint extensometers were installed after each step of the excavations, the displacements could not be recorded before the installation. However, the incremental displacements caused by the excavations of the slope below had been measured by the multipoint extensometers. Four incremental displacement measurements were selected for the back analysis: the third point of multipoint extensometer M^4^_1RX_ at EL 1135 m, the innermost point of the multipoint extensometer M^4^_2RX_ at EL 1165 m, the innermost point of the multipoint extensometer M^4^_1RJC_ at EL 1225 m, and the innermost point of the multipoint extensometer M^4^_2RJC_ at EL 1255 m. The last three excavation steps and their corresponding incremental displacements are shown in Table 2.

### 5.3. Training Sample Design

In elastic-plastic model, elastic modulus (*E*) has greater influence on rock mass deformation than Poisson ratio (*μ*), cohesion (*c*), and internal friction angle (*φ*) [25]. Therefore, the elastic moduli of the rock masses were the parameters to be back analyzed. In the completely weathered rocks, the tectonic stress had been released and had little influence on the deformation of the slope; the material parameters of the completely weathered rock were not involved in the back analysis. The elastic moduli of the four kinds of rock masses to be back analyzed are shown in Table 3, namely, *E*_1_, *E*_2_, *E*_3_, and *E*_4_. For the training samples, four different levels were selected in the range for each parameter in back analysis.

To establish the mapping function between the elastic moduli and incremental displacements, enough samples were designed for the BP network training. It is impossible to test all the combinations of the input and output parameters. Orthogonal experimental design has an ability to determine typical experimental schemes by considering complex combinations of many factors. Four factors and four levels were considered. Hence, the orthogonal table L_32_(4^9^) was designed. It meant that less than 9 factors and 4 levels could be considered, and the total number of combinations was 32.

The numerical simulations were conducted using the FLAC^3D^ code. An elastoplastic constitutive model and Mohr-Coulomb failure criterion were used in the simulations. Thus, 28 training samples and 4 testing samples were built for the back analysis, as shown in Table 4. For each sample, the input parameters were prepared by the orthogonal design method, while the corresponding output parameters were obtained by the numerical simulations.

### 5.4. BP Neural Network Initialized by PSO

The rock elastic moduli of the 4 zones were the input vectors. Two, three, and four monitoring incremental displacements were measured after the excavation Step 8, the excavation Step 9, and the excavation Step 10, respectively. And they constituted the output vectors separately as shown in Table 2. Namely, there were four nodes in the input layer of the BP neural network and two, three, or four in the output layer. As mentioned before, for a three-layer BP neural network with *M* nodes in input layer, if it has 2*M* + 1 nodes in the hidden layer and *N* nodes in the output layer, it can be trained to describe any function between the parameters [22]. For this reason, 3 BP network models were established with different network structures as follows: 4-9-2, 4-9-3 and 4-9-4.

The parameters of the PSO to be determined included particle number **m**, total evolution number **g** **e** **n**_**max**_, inertia weight **w**, and learning factors **c**_1_′ and **c**_2_′. Generally, **m** was set in the range of [20, 40] and **g** **e** **n**_**max**_ was a relatively large value according to the actual condition. An improved particle swarm optimization algorithm was proposed. The dynamic inertia weight **w** linearly decreased with the increasing of the iterative generation. Previous studies show that both the searching efficiency and precision of the PSO will increase greatly when the initial inertia weightis near 1.0 and the learning factors are near 2.0 [26].

**Figure 11:**
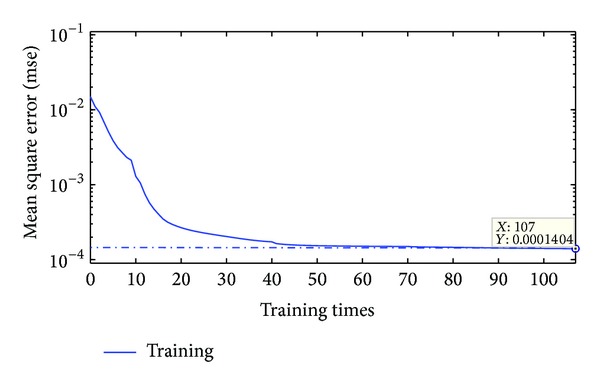
Convergence curve of the BP neural network.

The training samples were used to train the BP network, while the PSO was adopted to search for the relatively matched network model in advance, making the summed square error between the displacements from the numerical simulation and those from the BP network a minimum. Only the last neural network model was described here. In the particle swarm optimization model, the particle number was 25, the total evolution number was 200, the inertia weight was varied linearly from 0.95 to 0.4, the learning factors (**c**_1_′ and **c**_2_′) were both 2.02, and upper speed limit was 0.5. Then, the BP neural network initialized by PSO was trained by the trainlm algorithm. The tansig function and purelin function were used as the transfer functions from the input layer to the hidden layer and from the hidden layer to the output layer, respectively. The convergence curve for the summed square error is shown in Figure 11. The BP neural network was finally established for the following back analysis when the mean square error tended to be a minimal value.

### 5.5. Displacement Back Analysis

The elastic moduli of the rock masses are back analyzed using particle swarm optimization algorithm which had a global searching capability. For the excavation Step 10, PSO was applied to search for suitable parameter values according to the monitoring incremental displacements measured after Step 10 as shown in Table 2. The particle number was set to be 20, the total evolution number was 200, the inertia weight was varied linearly from 1.05 to 0.4, the learning factors (**c**_1_′ and **c**_2_′) were both 2.0, and the upper speed limit was set to be 0.5. The position vector of each particle had four dimensions, representing the four elastic moduli of the rock masses. When the fitness function reached a steady minimal value, the back-analyzed values of the elastic moduli of the rock masses in the right bank slope (*E*_1_, *E*_2_, *E*_3_, and *E*_4_) were obtained. The results showed that they were 0.45 GPa, 2.50 GPa, 2.76 GPa, and 7.98 GPa, respectively.

The back-analyzed elastic moduli were put into the numerical code to calculate the displacements at the monitoring points, and their elastic moduli also were used as the input in the trained BP neural network model to obtain the corresponding displacements at the monitoring points at different excavation steps. The displacements measured by the multipoint extensometers, the displacements simulated by the numerical code using the back-analyzed parameters, and the derived displacements using the BP neural networks are shown in Table 5.

It can be found that the predicted displacements of the BP neural network model using the back-analyzed elastic moduli as the input are close to the measured displacements in practical slope engineering.

The incremental displacements obtained by the BP neural network model and those measured at monitoring points of M^4^_2RJC_ and M^4^_1RJC_ after Step 8 and Step 10 were the same. The maximum absolute error between the predicted displacements and the measured displacements was −0.141 mm, and the relative errors were not greater than 20%. The calculated displacements by FLAC^3D^ using the estimated parameters were close to the measured ones. There results indicate that the parameters obtained by the back analysis were verified to be much reasonable for further slope stability analysis.

The slope stability after excavating Step 10 was analyzed using FLAC code by assigning the estimated elastic moduli to the four kinds of rock masses. The strength reduction method was applied in the numerical simulation, by reducing the strength of the rock masses gradually step by step to obtain the safety factor of the slope.

**Figure 12:**
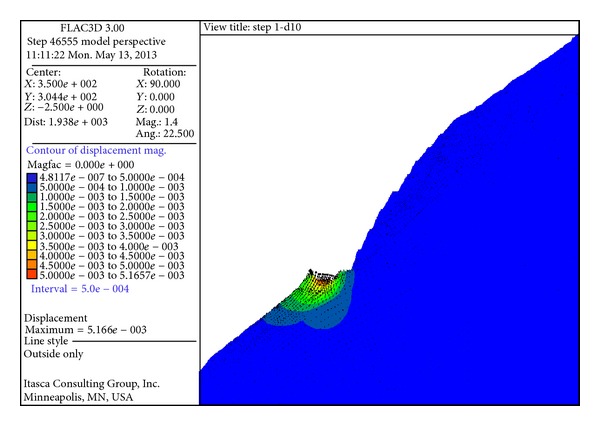
Displacement contour and displacement vector distribution after step 10 (unit: m).

**Figure 13:**
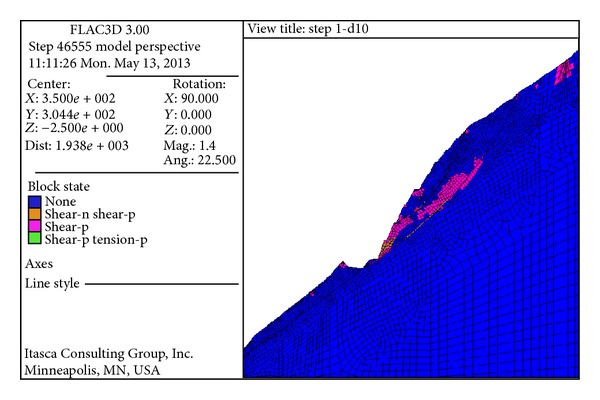
Failure state of the slope model.

Figure 12 shows the slope displacement distribution after excavating Step 10. The phenomena of ground heave appeared caused by the excavation and geostress unloading at EL 1100 m.  Figure 13 shows the failure state of the slope model. The blue blocks were in elastic state, and blocks in the other colors were the plastic zone. It can be found that the plastic zones develop downward along the fissures XL_316-1_, especially in the area between fissures XL_316-1_ and XL_9-15_. The large plastic zone from EL 1135 to EL 1195 had considerable influence on the slope stability.

## 6. Conclusions

The BP neural network model was proposed to estimate the elastic moduli of the rock masses of the right bank high slope in Dagangshan Hydroelectric Power Station. The particle swarm optimization model was applied to optimize the BP neural network. The displacements at the monitoring points were obtained by putting the back-analyzed parameters into the numerical simulations and the BP neural network. The major findings and conclusions can be summarized as follows.

1. The BP neural network and the particle swarm optimization model were successfully applied in the deformation parameter estimation of the slope rock masses in Dagangshan Hydroelectric Power Station. The BP network model was established to express the highly nonlinear mapping relation between the mechanical parameters and deformation behaviors of the four rock masses in complex stress state disturbed by the excavations. The particle swarm optimization model helped the BP network initialize the weights and thresholds and search for the suitable values of the elastic moduli within the large parameter space. The results showed that the parameters were reasonable and acceptable in the slope stability analysis. The displacement back analysis based on the BP neural network and the particle swarm optimization was proved to be an efficient and reliable method for parameter estimation of rock masses under complex geological conditions and large-scale excavations.
2. To avoid the loss of instantaneous displacement, the incremental displacements at each excavation step were used to back-analyze the elastic moduli of the rock masses. As the multipoint extensometers were installed after each step of the excavations, the displacements of the rock mass caused by elastic strain release before that step could not be measured. The incremental displacements of the rock masses measured by the multipoint extensometers were adopted for back analysis.
3. The back-analyzed parameters were put into the numerical model to obtain the displacements of the monitoring points. The displacements simulated by the numerical model were compared with the measured displacement and the output displacements by the BP network using the back-analyzed parameters. This provided a potentially useful way to examine the validity of the previously established BP network model.
4. The estimated parameters were used in the numerical model to predict the stability of the slope by using the strength reduction method. The numerical analysis indicated that the stability of the right bank slope is controlled by a potentially instable block whose slip surfaces were formed by many structural planes including the unloading fissures XL_316-1_ and the fault *f*_231_.

## Figures and Tables

**Table 1:** Parameters employed in the numerical simulations.

Rock mass	*g* (g*·*cm^−3^)	*σ*_*c*_ (MPa)	*E* (GPa)	*μ*	*c* (MPa)	*ϕ* (°)
II	2.65	70~80	18.00~25.00	0.25	2.000	52.43
III_1_	2.62	40~60	9.00~11.00	0.27	1.500	50.19
III_2_	2.62	40~60	6.00~9.00	0.30	1.000	45.00
IV	2.58	20~40	2.50~3.50	0.35	0.700	38.66
V_1_	2.45	<15	0.25~0.50	>0.35	0.500	33.02
V_2_	2.10	<10	0.20	>0.35	0.300	26.57

**Table 2:** Measured incremental displacement.

Excavation step	M^4^_2RJC_	M^4^_1RJ_	M^4^_2RX_	M^4^_1RX_
Step 8	0.82 mm	0.87 mm	—	—
Step 9	1.35 mm	1.37 mm	2.30 mm	—
Step 10	0.15 mm	0.12 mm	−0.01 mm	−0.10 mm

**Table 3:** Elastic modulus levels of the four types of the slope rock masses.

Zone	Elastic modulus (GPa)	Level (GPa)
I (*E*_1_)	[0.25, 0.50]	0.25, 0.33, 0.42, 0.50
II (*E*_2_)	[2.50, 3.50]	2.50, 2.80, 3.20, 3.50
III (*E*_3_)	[2.50, 3.50]	2.50, 2.80, 3.20, 3.50
IV (*E*_4_)	[6.00, 9.00]	6.00, 7.00, 8.00, 9.00

**Table 4:** The incremental displacements of the monitoring points in the orthogonal experimental design.

ID	Elastic modulus (GPa)				Incremental displacement (mm)								
					Step 8		Step 9			Step 10			
	Zone I	Zone II	Zone III	Zone IV	M^4^_2RJC_	M^4^_1RJC_	M^4^_2RJC_	M^4^_1RJC_	M^4^_2RX_	M^4^_2RJC_	M^4^_1RJC_	M^4^_2RX_	M^4^_1RX_
1	0.25	2.5	2.5	6	0.38	0.28	1.35	1.64	2.22	0.09	0.08	−0.01	−0.11
2	0.25	2.8	2.8	7	0.46	0.39	2.39	2.73	2.31	−0.09	−0.08	−0.06	−0.07
3	0.25	3.2	3.2	8	0.45	0.39	1.35	1.64	2.22	0.12	0.14	−0.01	−0.08
⋮	*⋯*	*⋯*	*⋯*	*⋯*	*⋯*	*⋯*	*⋯*	*⋯*	*⋯*	*⋯*	*⋯*	*⋯*	*⋯*
30	0.5	2.8	3.5	8	0.24	0.08	1.81	2.25	1.58	0.13	0.13	−0.08	−0.09
31	0.50	3.2	2.5	7	0.85	0.88	1.65	1.99	2.61	0.11	0.11	0.02	−0.08
32	0.50	3.5	2.8	6	0.51	0.50	1.19	1.50	2.29	−0.00	−0.01	−0.07	−0.09

**Table 5:** Comparison of back analysis values, BP values, and measured values.

Excavation step	Monitoring point	Increment displacement (mm)		
		FLAC	BP	Measurement
Step 8	M^4^_2RJC_	0.869	0.820	0.820
	M^4^_1RJC_	0.943	0.870	0.870

Step 9	M^4^_2RJC_	1.456	1.240	1.350
	M^4^_1RJC_	1.775	1.511	1.370
	M^4^_2RX_	2.386	2.285	2.300

Step 10	M^4^_2RJC_	0.138	0.131	0.150
	M^4^_1RJC_	0.143	0.143	0.120
	M^4^_2RX_	−0.012	−0.010	−0.010
	M^4^_1RX_	−0.078	−0.100	−0.100
